# Pathogenicity and identification of host adaptation genes of the avian pathogenic *Escherichia coli* O145 in duck

**DOI:** 10.3389/fcimb.2024.1453907

**Published:** 2024-11-13

**Authors:** Mei-Fang Tan, Jia Tan, Shao-Pei Fang, Zhao-Feng Kang, Hai-Qin Li, Fan-Fan Zhang, Cheng-Cheng Wu, Na Li, Yan-Bin Zeng, Cui Lin, Jiang-Nan Huang

**Affiliations:** ^1^ Institute of Animal Husbandry and Veterinary Science, Jiangxi Academy of Agricultural Sciences, Nanchang, China; ^2^ Jiangxi Province Key Laboratory of Animal Green and Healthy Breeding, Nanchang, China

**Keywords:** avian pathogenic *Escherichia coli*, O145, pathogenicity, host adaptation, metabolic pathway, duck

## Abstract

**Introduction:**

Avian pathogenic *Escherichia coli* (APEC) is a critical bacterial pathogen that causes severe infections in poultry. Diverse serotypes increase the complexity of treatment and controlling APEC infections. Recent epidemiological investigations indicate O145 is emerging as a predominant serogroup of APEC in China. However, limited information is known about this newly emerged serogroup.

**Methods:**

A virulent strain, NC22, was selected to elucidate the mechanisms underlying APEC O145-related pathogenicity and host adaptation. Whole-genome sequencing and pathogenicity assays was conducted on this strain. We further performed a transcriptional analysis of the bacteria during the early colonization stage in the duck liver and compared them with those in liquid cultures *in vitro*.

**Results:**

Subcutaneous inoculation of NC22 induced typical symptoms in ducks. The bacterial loads in the blood and various tissues peaked at 2 and 3 days post infection, respectively. The affected tissues included the heart, liver, spleen, lung, kidney, bursa of Fabricius, duodenum, jejunum, and cecum. We then analyzed the transcriptome profiles of NC22 during growth in duck liver versus lysogeny broth and identified 87 genes with differential expression levels.These included key metabolic enzymes and recognized host adaptation factors. Analysis of the metabolic pathways revealed an inhibition of the metabolic shift from glycolysis towards pentose phosphate pathway and an interference of the citrate cycle. Moreover, significantly differentially expressed small regulatory RNAs were examined, such as SroC, CsrC, and GadY.

**Discussion:**

These findings enhance our understanding of the pathogenicity of APEC O145 and the molecular mechanisms underlying APEC-related pathogen−host interactions.

## Introduction

Avian pathogenic *Escherichia coli* (APEC) is a causative bacterial pathogen in the poultry industry that causes disease in birds, such as chickens, ducks, and geese (Hu et al., 2022). Avian colibacillosis results in high morbidity and mortality due to systemic infections, including colisepticemia, pericarditis, perihepatitis, and peritonitis (Joseph et al., 2023). This bacterial disease is widespread and highly damaging to the poultry industry on a global scale (Joseph et al., 2023). Additionally, APEC potentially serves as a reservoir of virulence and resistance genes for human extraintestinal pathogenic *E. coli* (Bauchart et al., 2010). This zoonotic potential poses a risk to human health through environmental exposure and the consumption of animal-derived food products (Li et al., 2020). The effective prevention and control of APEC in poultry flocks is crucial for sustainable poultry production and public health (Joseph et al., 2023).

Although the pathogenic mechanism, antibiotic resistance, and vaccines of APEC strains have been extensively studied (Kathayat et al., 2021; Hu et al., 2022), diverse serotypes increases the complexity of treatment and controlling APEC infections. Among the numerous serotypes of APEC, O1, O2, and O78 have traditionally been considered the predominant serogroups worldwide (Kathayat et al., 2021). Recent epidemiological investigations indicate O145 is emerging as a predominant serogroup of APEC in China and have stronger pathogenicity (Wang et al., 2022, 2023). The discovery of the ubiquity of O145 may explain the failure of vaccines that target the three major serogroups (Wang et al., 2022). However, limited information is available about this newly emerged serogroup.

Pathogenic bacteria have developed effective stress response mechanisms to adapt to challenging *in vivo* environments (Zhang et al., 2016). The ability of APEC strains to survive and grow in the host is a key virulence determinant and contributes significantly to the pathogenesis of avian colibacillosis (Li et al., 2011). A chicken infection model by APEC O78 showed dozens of genes involved in adhesin, lipopolysaccharide core synthesis, and iron-responsive system are pathogen-specific factors expressed in air sacs or pericardium (Dozois et al., 2003). Transcriptome analysis revealed that a series of genes involved in adaptive metabolism, protein transport, biosynthesis pathways, and stress resistance contribute to the adaptation and growth of APEC O1 in chicken serum (Li et al., 2011). Genome-wide analysis revealed that more than one hundred host adaptation genes of extraintestinal pathogenic *E. coli* O2 are required for colisepticemia in mammalian (mouse) and avian (duck) models (Zhang et al., 2019).

In this study, a strain, NC22, that caused severe infection in ducks was used to study the pathogenic characteristics and *in vivo* adaptability of APEC O145. We conducted whole-genome sequencing and pathogenicity assays on this strain. Subsequently, we performed a transcriptional analysis of the bacteria during the early colonization stage in the duck liver and compared them with those in liquid cultures *in vitro*. Differentially expressed genes (DEGs) encoding important metabolic enzymes and known host adaptation factors were identified, as well as differently expressed small regulatory RNAs (sRNAs). These results can enhance our understanding of the pathogenicity of APEC O145 and the molecular mechanisms underlying APEC-related pathogen−host interactions.

## Materials and methods

### Bacterial strains and growth conditions

The virulent APEC O145 strain NC22 used in this study was isolated from Tianfu duck flocks suffering from APEC infections in a large duck farm in Nanchang city, China, in 2022 (Tan et al., 2023). Bacteria were obtained from brain and liver samples. Bacterial identification and antimicrobial susceptibility had been conducted (Tan et al., 2023). The APEC strain was grown at 37°C in lysogeny broth (LB; Hopebio, Qingdao, China) and on LB agar (Hopebio) plates.

### Genomic sequencing, annotation, and analysis

The genomic DNA of NC22 was extracted using a MiniBEST Bacteria Genomic DNA Extraction Kit (TaKaRa, Dalian, China) following the manufacturer’s instructions. Whole-genome sequencing was conducted using the Oxford Nanopore Technologies platform and Illumina MiSeq platform at Guangzhou Genedenovo Biotechnology (China). Sequencing reads were assembled using Flye software (version 2.9.3) and validated using Pilon software (version 1.23). Automated genome annotation was carried out using Prokka (Seemann, 2014). Predicted genes were further annotated through BLAST search against different bioinformation databases at a cut-off E-value of 1.0E^−5^, including the NCBI nonredundant protein database, UniProt/Swiss Prot, Kyoto Encyclopedia of Genes and Genomes (KEGG), Gene ontology, Clusters of orthologous groups of proteins, and Protein families database.

The complete genome and plasmid sequences of NC22 were deposited in GenBank and the accession numbers are listed in **Table 1**. The genotype of NC22 were confirmed by uploading the whole genome sequence and conducting online analysis on the Center for Genomic Epidemiology (CGE) website (http://www.genomicepidemiology.org/services/). The acquired antibiotic resistance genes of NC22 were identified using the ResFinder platform (version 4.4.3) in CGE. The virulence genes of NC22 were predicted using the VirulenceFinder platform (version 2.0.5) in CGE.

**Table 1:** Genome characteristics of the NC22 strain.

Location	Size (bp)	GC content	No. of genes	GenBank accession No.	Acquired resistance genes	Virulence genes
Chromosome	4886732	50.64%	4426	CP109953	*aph(3’’)-Ib*, *aph(6)-Id*, *bla*_CTX-M-55_, *floR*, *sul2*, *tet(A)*	*csgA*, *fdeC*, *fimH*, *fyuA*, *gad*, *hha*, *hlyE, irp2*, *iss*, *lpfA*, *neuC*, *nlpI*, *ompT*, *papA*_*F19*, *shiA*, *terC*, *tia*, *yehABCD*
Plasmid p1	7136	48.64%	10	CP109954	−	−
Plasmid p2	234214	46.66%	234	CP109955	*aac(3)-IIa*, *aadA16*, *aph(3’)-Ia*, *arr-3*, *bla*_CTX-M-64_, *dfrA27*, *mphA*, *oqxA*, *oqxB*, *qacE*, *sul1*, *tet*(A)	*terC*
Plasmid p3	255823	49.28%	219	CP109956	−	*anr*, *cvaC*, *etsC*, *hlyF*, *iroN*, *iss*, *iucC*, *iutA*, *mchF*, *ompT*, *sitA*, *traJ*, *tsh*

### Electron microscopy

The NC22 strain was cultured in LB medium at 37°C and harvested during the logarithmic growth period (at an optical density of 600 nm of ~0.8). Bacteria were fixed with 2.5% glutaraldehyde at 4°C overnight. Scanning electron microscopy (SEM) and transmission electron microscopy (TEM) analyses were performed and observed in accordance with previously described methods (Tan et al., 2022).

### Bacterial inoculation assays

To assess the pathogenicity of NC22 in ducks, a total of 60 female 7-day-old Tianfu ducks (10 ducks per group) were subcutaneously infected with bacteria in the mid-log phase. One-day-old specific pathogen-free ducks were obtained from Shangdong Health-tech Laboratory Animal Breeding Co., Ltd. (China) and housed in isolators until use. The inoculation amounts for each group were 2.8×10^9^, 2.8×10^8^, 2.8×10^7^, 2.8×10^6^, and 2.8×10^5^ CFU/duck, respectively. Ducks in the negative control group were inoculated with an equal amount of physiological saline. The infected ducks were monitored for 7 days. Pathological dissection was performed on the deceased ducks. Survival curves were generated using GraphPad Prism 8.0 software (San Diego, USA). The median lethal dose (LD_50_) of NC22 for ducks was calculated by the method of Probits using IBM SPSS version 26.0 software (New York, USA).

### *In vivo* colonization assay

To evaluate the ability of NC22 to colonize in the bloodstream and various tissues in ducks, a total of 30 7-day-old female Tianfu ducks were subcutaneously injected with 1.36×10^7^ CFU/duck of bacteria in the mid-log phase. Blood, heart, liver, kidney, spleen, lung, kidney, and brain samples were obtained from three ducks at each time point (1, 2, 3, 5, and 7 days post infection (dpi)). The blood and various tissue homogenates in sterile saline were plated onto LB agar plates, and the colonization of bacteria was determined as previously described (Horn et al., 2012). Additionally, tissue samples from the trachea, liver, heart, spleen, lung, kidney, bursa of Fabricius, thymus, pancreas, duodenum, jejunum, ileum, rectum, cecum, and brain were collected at 1, 2, and 3 dpi and fixed in 4% paraformaldehyde for 2-3 days. The samples were then embedded in paraffin wax, cut into sections, stained with hematoxylin and eosin, and examined for lesions by light microscopy at Wuhan Servicebio Technology Co., Ltd (China) as described previously (Horn et al., 2012).

### Transcriptome sequencing and analysis

During the *in vivo* colonization assay, the liver tissues of infected ducks at 2 dpi were collected. Bacteria grown to the log phase in LB medium served as the reference sample. Bacterial RNA was obtained from the liver tissues and liquid cultures using the SV Total RNA Isolation System (Promega, Wisconsin, USA) according to the manufacturer’s instructions. The quality and concentration of RNA were monitored using a BioPhotometer plus (Eppendorf, Hamburg, Germany).

RNA sequencing was performed at Guangzhou Genedenovo Biotechnology. Library preparation, sequencing, quality control, and read mapping to the reference genome were conducted as previously described (Tan et al., 2022). EdgeR (version 3.10.2) was used to analyze differences in gene expression. The main parameters were set as follows: log_2_|fold change| > 1 and a false discovery rate (FDR) < 0.05. FDR was used in multiple hypothesis testing to correct for the *P* value (Zhang et al., 2017). Enrichment analysis was performed based on the KEGG database using the Blast2GO software (https://www.blast2go.com/).

The expression level of bacterial sRNAs was calculated using the transcript per million method based on previous research (Bao et al., 2019). sRNAs with an absolute log value ≥ 2 and an FDR value < 0.05 were considered to be significantly changed in response to colonization within the host. Candidate sRNAs were annotated using BLAST in the sRNAMap database (http://srnamap.mbc.nctu.edu.tw/).

### Quantitative real-time PCR

A subgroup consisting of 8 DEGs was selected to validate the reliability of the transcriptome data using qRT-PCR. The internal reference gene used was 16S rDNA. Nine pairs of primers (**Supplementary Table S1**) were designed based on the genomic sequence of the NC22 strain. The cDNA library established during transcriptome sequencing served as the template for qRT-PCR, which was performed using the AceQ qPCR SYBR Green Master Mix (Vazyme, Nanjing, China) on an ABI Prism 7900HT Sequence Detection System. The relative expression level was calculated using the 2^−ΔΔCt^ method (Livak and Schmittgen, 2001). The data are presented as the means ± standard deviations of the bacteria in liver samples and liquid cultures.

### Ethics statement

Approval from the Animal Ethics Committee of the Institute of Animal Husbandry and Veterinary Medicine, Jiangxi Academy of Agricultural Science (2010-JXAAS-XM-01), was obtained for the collection of blood and tissue samples from the experimental ducks. All efforts were made to minimize suffering.

## Results

### Characterization of the NC22 strain

**Figure 1:**
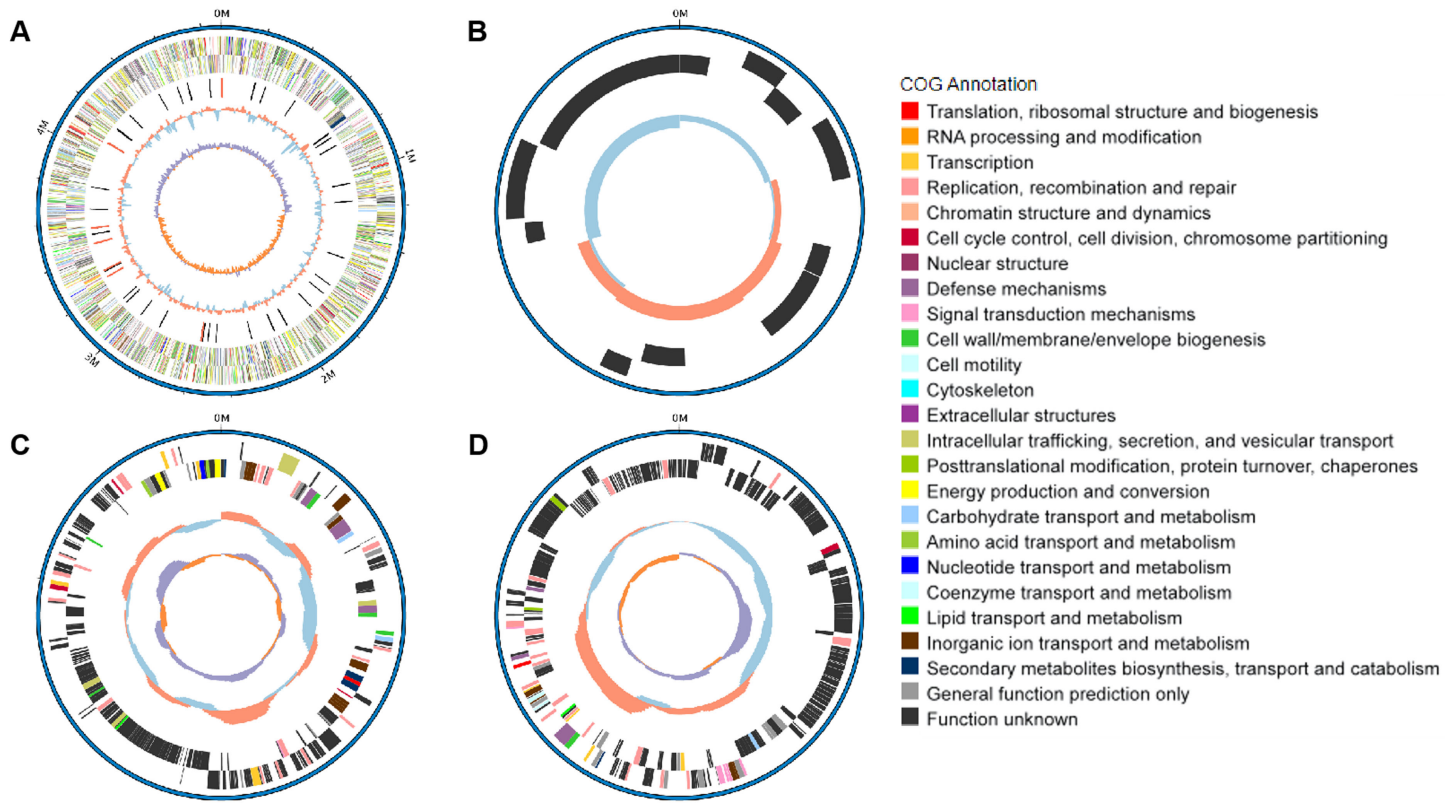
Genomic features and functional annotation of the chromosome **(A)**, plasmid P1 **(B)**, plasmid P2 **(C)**, and plasmid P3 **(D)** of NC22.

The serotype of NC22 was identified as O145:H9. Whole-genome sequencing revealed that the genome of NC22 contains a single chromosome (**Figure 1A**) and three plasmids (**Figures 1B–D**) with sizes of 4.89 Mbp and 7,136, 234,214, and 255, 823 bp (**Table 1**), respectively. The chromosome contained 4,426 coding sequences, with an average G + C content of 50.26%. The chromosome harbored six acquired antibiotic resistance genes, including the aminoglycoside resistance genes *aph(3’’)-Ib* and *aph(6)-Id* and the β*-*lactamase resistance gene *bla*_CTX-M-55_ (**Table 1**; **Supplementary Table S2**). The chromosome also contained 21 virulence genes, such as the adhesion factor *fimH*, protectin gene *ompT*, and toxin *hlyE* (**Table 1**; **Supplementary Table S3**). Three circular plasmids were detected in the strain, with plasmid P2 carrying 12 acquired antibiotic resistance genes (**Table 1**; **Supplementary Table S2**) and plasmid P3 carrying 13 virulence genes (**Table 1**; **Supplementary Table S3**).

**Figure 2:**
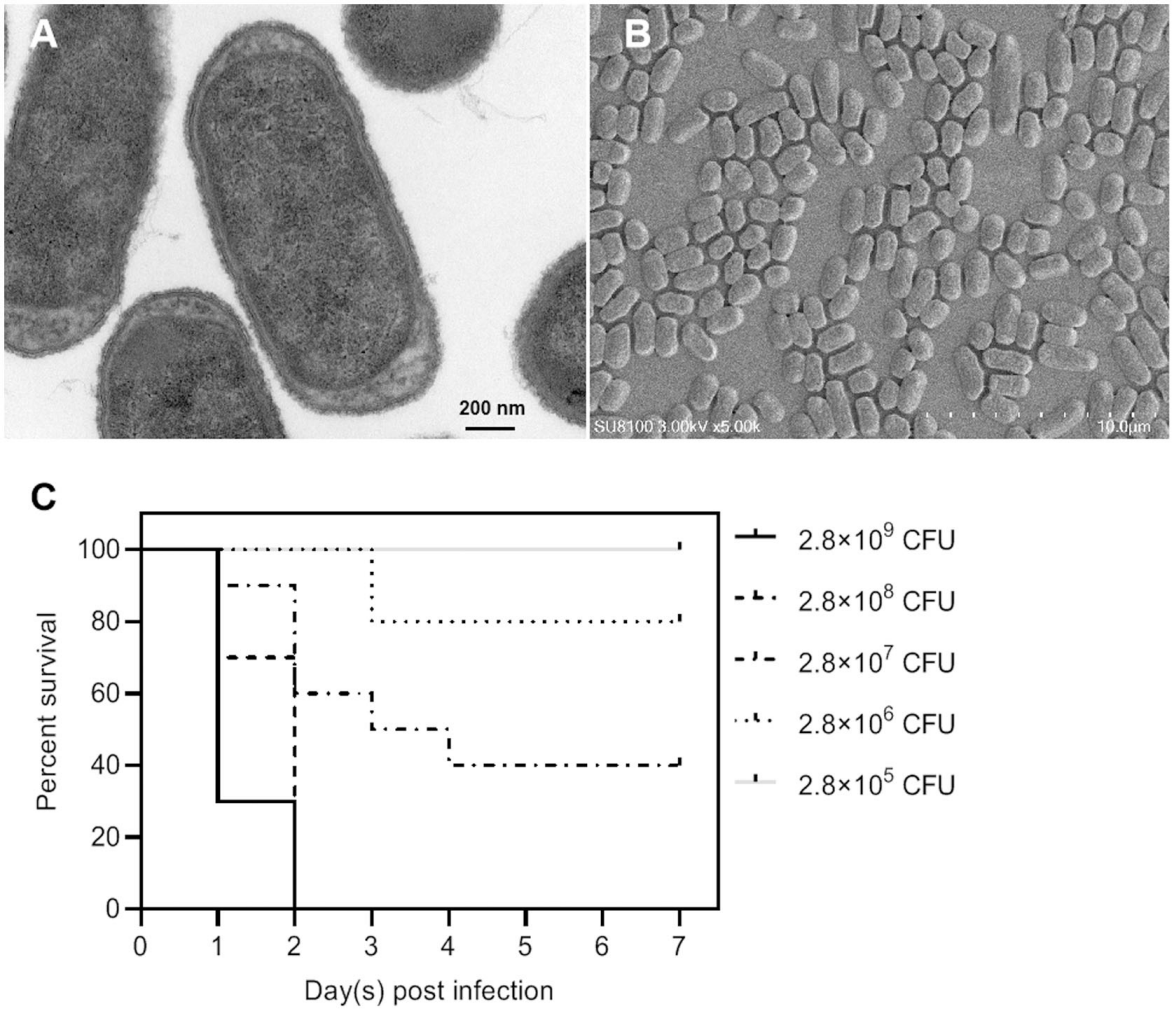
Morphological observation and pathogenicity of the NC22 strain. **(A)** Transmission electron micrographs of the bacteria. **(B)** Scanning electron micrographs of the bacteria. **(C)** Survival curves of NC22-challenged ducks. The survival rate of the ducks in the negative control group was 100%, which is not shown in the figure.

Among all these virulence genes, *iss*, *tsh*, *iutA*, *cvaC*, *hlyF*, *iroN*, *ompT*, and *iutA* are typical virulence factors that can be used to distinguish APEC from other types of *E. coli* (Ovi et al., 2023), indicating that NC22 is an APEC strain. Additionally, the bacterial morphology of NC22 were observed through electron micrographs of SEM (**Figure 2A**) and TEM (**Figure 2B**).

### Survival rate and clinical signs of APEC-infected ducks

The pathogenicity of NC22 was assessed using infection models involving 7-day-old Tianfu ducks. Five groups of ducks were injected with various dilutions of bacteria *via* subcutaneous infection, while physiological saline was used as a negative control. The survival of each group is shown in **Figure 2C**. All ducks died within 2 days at infection doses of 2.8×10^9^ and 2.8×10^8^ CFU/duck. All the ducks survived and appeared healthy at the end of the observation period when infected with a dose of 2.8×10^5^ CFU/duck. Based on the percent survival data, the LD_50_ of NC22 for Tianfu ducks was calculated to be 1.36×10^7^ CFU.

Clinical symptoms were observed on the ducks infected with high infection doses, including fever, rhinorrhea, depression, and diarrhea. Necropsy examination of dying ducks revealed severe fibrous exudate on the surface of the peritoneum, liver, and heart (**Supplementary Figure S1**) and the damage of multiple organs. Conversely, no significant pathological changes were observed in the various tissues of the control group.

### Bacterial colonization ability *in vivo*

Ducks were subcutaneously vaccinated with bacteria at a dose of 1×LD_50_. Bacteria were subsequently recovered from the blood (**Figure 2A**), heart (**Figure 2B**), liver (**Figure 2C**), spleen (**Figure 2D**), lung (**Figure 2E**), kidney (**Figure 2F**), and brain (**Figure 2G**) at different time points postinfection. The results revealed rapid replication of APEC in the blood and all tested tissues at 1 dpi. The bacterial load in the blood peaked at 2 dpi. Peak bacterial loads in the parenchymal tissues were observed at 3 dpi. By 7 dpi, the bacteria in the blood, heart, liver, spleen, and kidney had been completely eliminated. However, bacteria were still detected in the lung and brain at 7 dpi.

### Pathohistological observation of infected ducks

**Figure 3:**
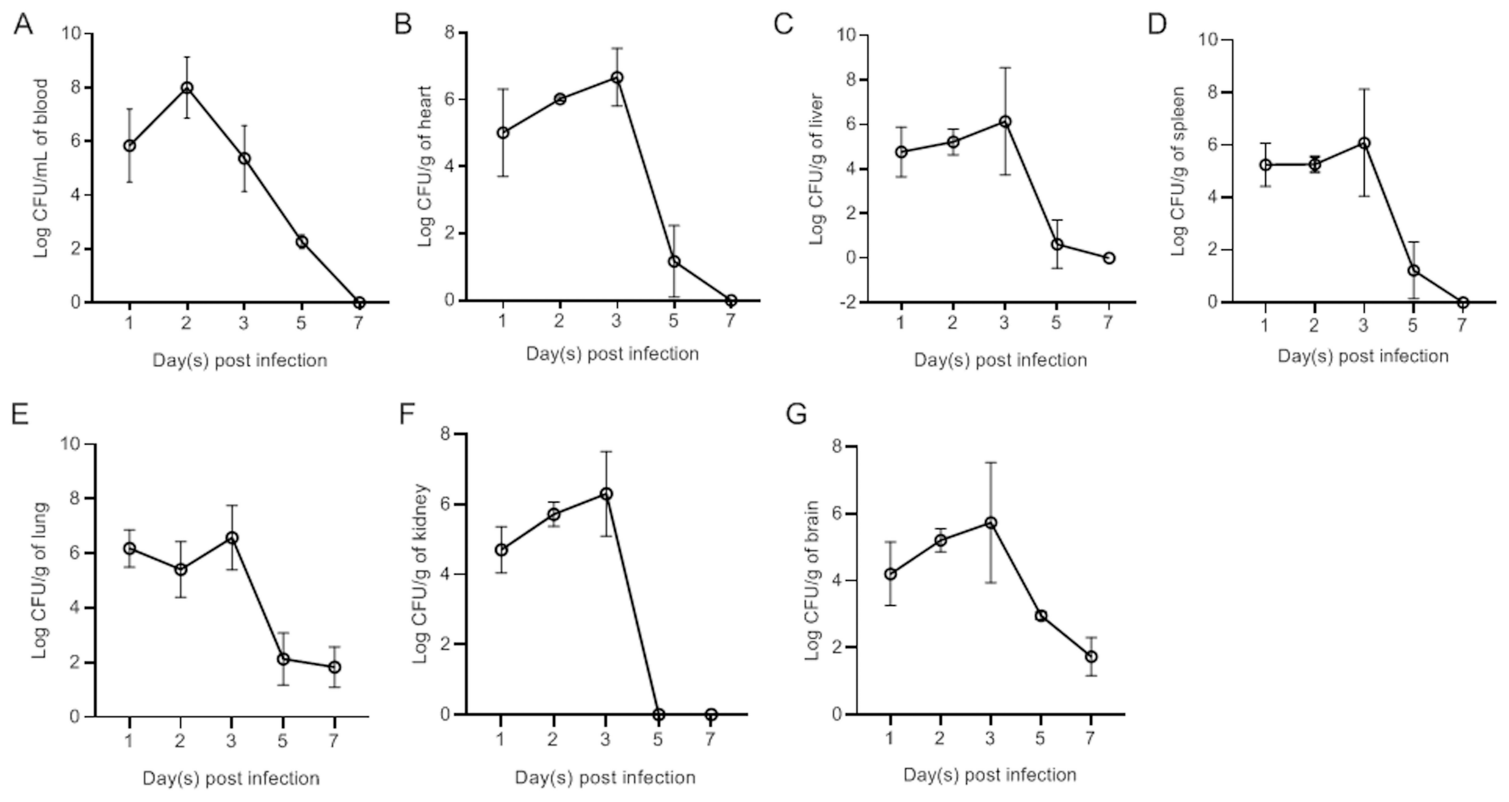
Bacterial loads in the blood **(A)**, heart **(B)**, liver **(C)**, spleen **(D)**, lung **(E)**, kidney **(F)**, and brain **(G)** of NC22-challenged ducks. The data are expressed as the mean ± SEM.

To investigate the tissue lesions caused by NC22 infection in ducks, a total of 15 various tissues were acquired daily during the first three days following infection, which was when the bacterial loads were abundant *in vivo*. The tissues associated with the observed lesions included the heart, liver, spleen, lung, kidney, bursa of Fabricius, duodenum, jejunum, and cecum (**Figure 3**). No lesions were observed in the trachea, thymus, pancreas, ileum, rectum, or brain parenchyma. The various pathological symptoms of each tissue, along with the corresponding timing of occurrence, are summarized and listed in **Supplementary Table S4**.

**Figure 4:**
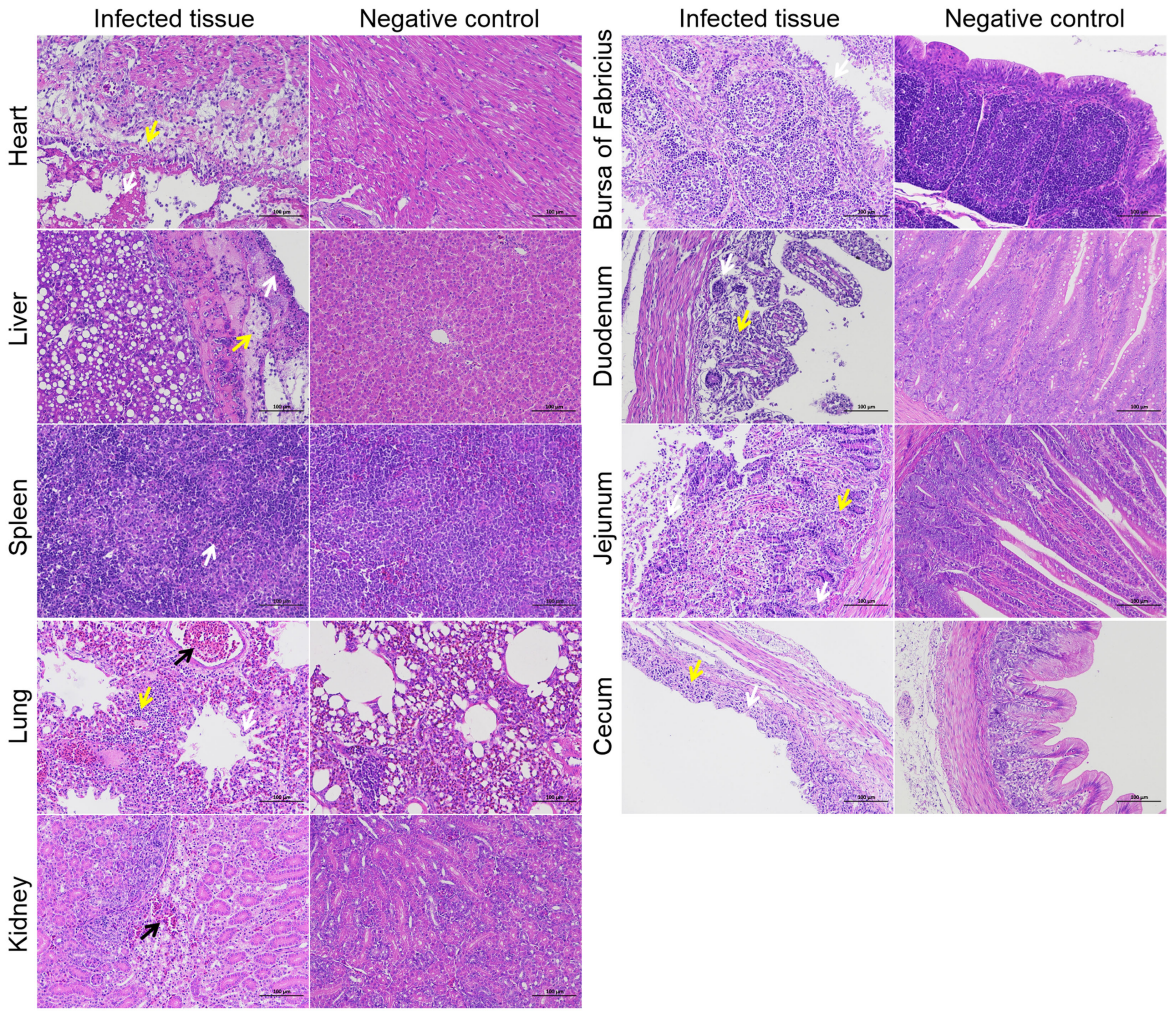
Histopathological examination of specific tissues at 3 dpi. The yellow arrows represent inflammatory infiltration. The white arrows represent cell or structural necrosis. The black arrows represent tissue hemorrhages. Bars, 100 μm.

Necrosis of myocardial cells, liver membranes, splenic parenchymal cells, renal tubular epithelial cells, intestinal glands, and intestinal villous epithelial cells was observed as a result of bacterial infection, as depicted in **Figure 4**. Bleeding symptoms were observed in the liver, lung, and kidney. Congestion symptoms were observed in the liver and kidney at 3 dpi, while severe bleeding occurred in the pulmonary accessory bronchi at 1 dpi. Inflammatory cell aggregation was observed in the heart, liver, lung, duodenum, jejunum, and cecum. Additionally, a significant decrease in lymphocytes in the duck bursa of Fabricius was observed after infection. These pathological observations indicate that the bacteria extensively attack specific tissues of the ducks.

### Transcriptomic analysis of bacteria *in vivo*

To investigate the transcriptional response of APEC to colonization within host tissue, we compared the bacterial transcriptomic profiles of infected liver tissues at 2 dpi with those of log phase liquid cultures of NC22. Using a > 2.0-fold change cutoff, we identified 87 DEGs (**Supplementary Table S5**), which accounted for 1.97% (87/4426) of the whole genome. Among these DEGs, 26 genes were upregulated, and 61 genes were downregulated.

From the RNA sequencing data, we selected three upregulated and five downregulated genes with different transcription levels for qRT−PCR analysis (**Table 2**). The correlation between RNA sequencing and qRT−PCR results was high (R^2^ = 0.8734; **Supplementary Figure S2**), confirming the reliability of the transcriptomic data in this study.

**Table 2:** Validation of RNA-Seq results by real-time quantitative PCR (qRT−PCR).

Gene locus tag	Gene	Description	Fold change of RNA-Seq	Fold change of qRT-PCR
OI124_00680	*tktB*	Transketolase	-30.88	-47.76 ± 3.03
OI124_04745	*katE*	Catalase HPII	-19.32	-12.50 ± 1.40
OI124_06060	*gadC*	L-glutamate:4-aminobutyrate antiporter	-21.25	-16.15 ± 3.51
OI124_07700	*phoQ*	Sensor protein	4.38	3.96 ± 0.65
OI124_08705	*ompF*	Porin	2.21	5.38 ± 1.12
OI124_15285	*fdhF*	Formate dehydrogenase H	5.69	5.06 ± 1.01
OI124_15965	*argC*	N-acetylglutamylphosphate reductase	9.73	13.47 ± 2.86
OI124_18540	*hdeA*	Acid-resistance protein	-5.13	-7.25 ± 1.04

**Figure 5:**
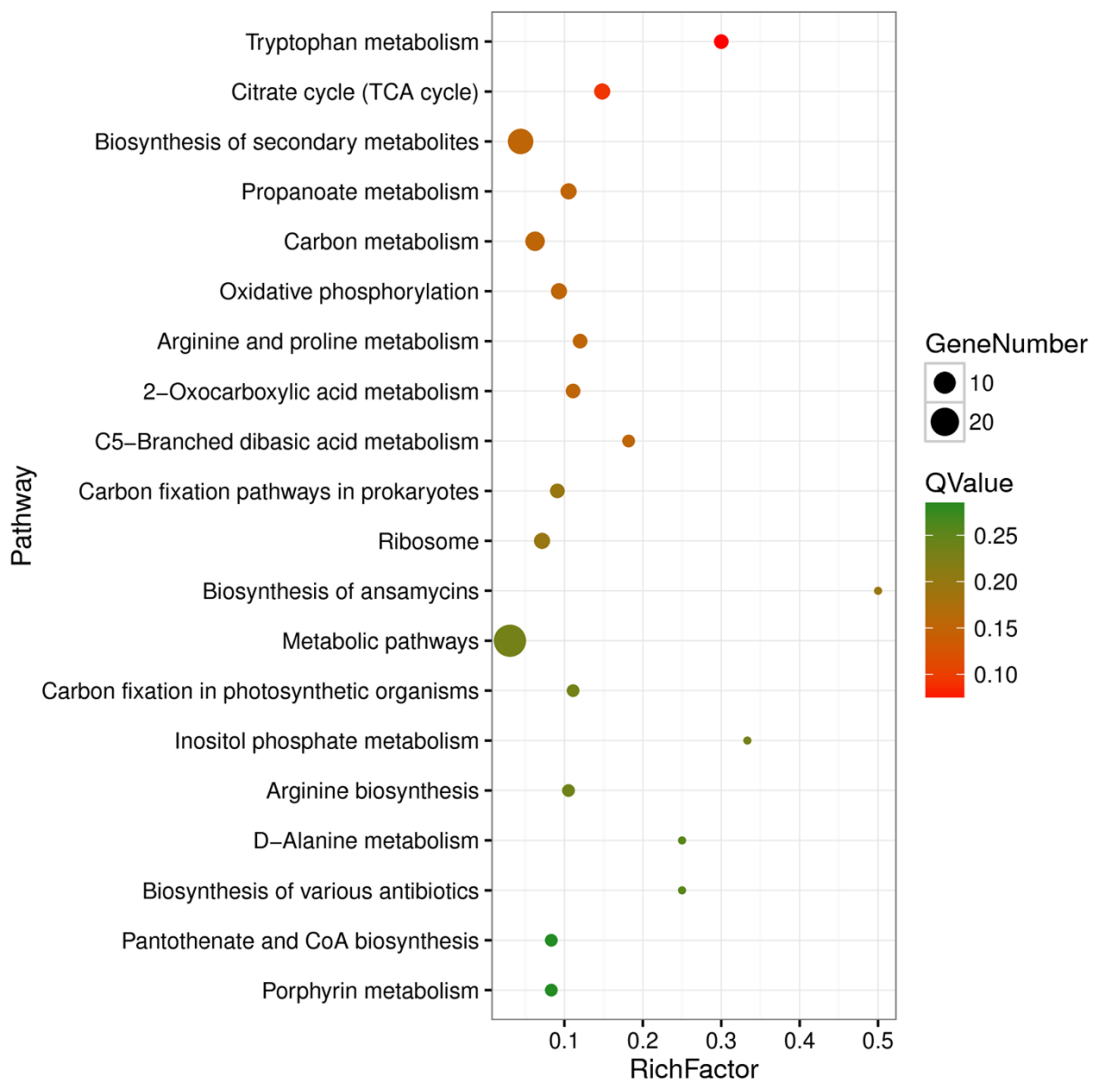
Top 20 enriched KEGG pathways of differentially expressed genes. Rich factor refers to the ratio of the number of differentially expressed genes enriched in the pathway to the number of all genes annotated in the pathway.

Functional enrichment analysis based on the KEGG database revealed that the DEGs were enriched in 48 KEGG pathways, with pathways belonging to the “Metabolism” class accounting for 81.25% (39/48) of the DEGs (**Supplementary Table S6**). The significantly enriched pathways included tryptophan metabolism, citrate cycle (TCA cycle), biosynthesis of secondary metabolites, propanoate metabolism, carbon metabolism, oxidative phosphorylation, and arginine and proline metabolism (**Supplementary Table S6**; **Figure 5**).

### DEGs in the central metabolic pathways

During the initial phase of infection in the host liver, there were notable alterations in the transcriptional levels of several key metabolic enzymes involved in central metabolic pathways of APEC, such as the pentose phosphate pathway (PPP) and the TCA cycle (**Figure 5**).

**Figure 6:**
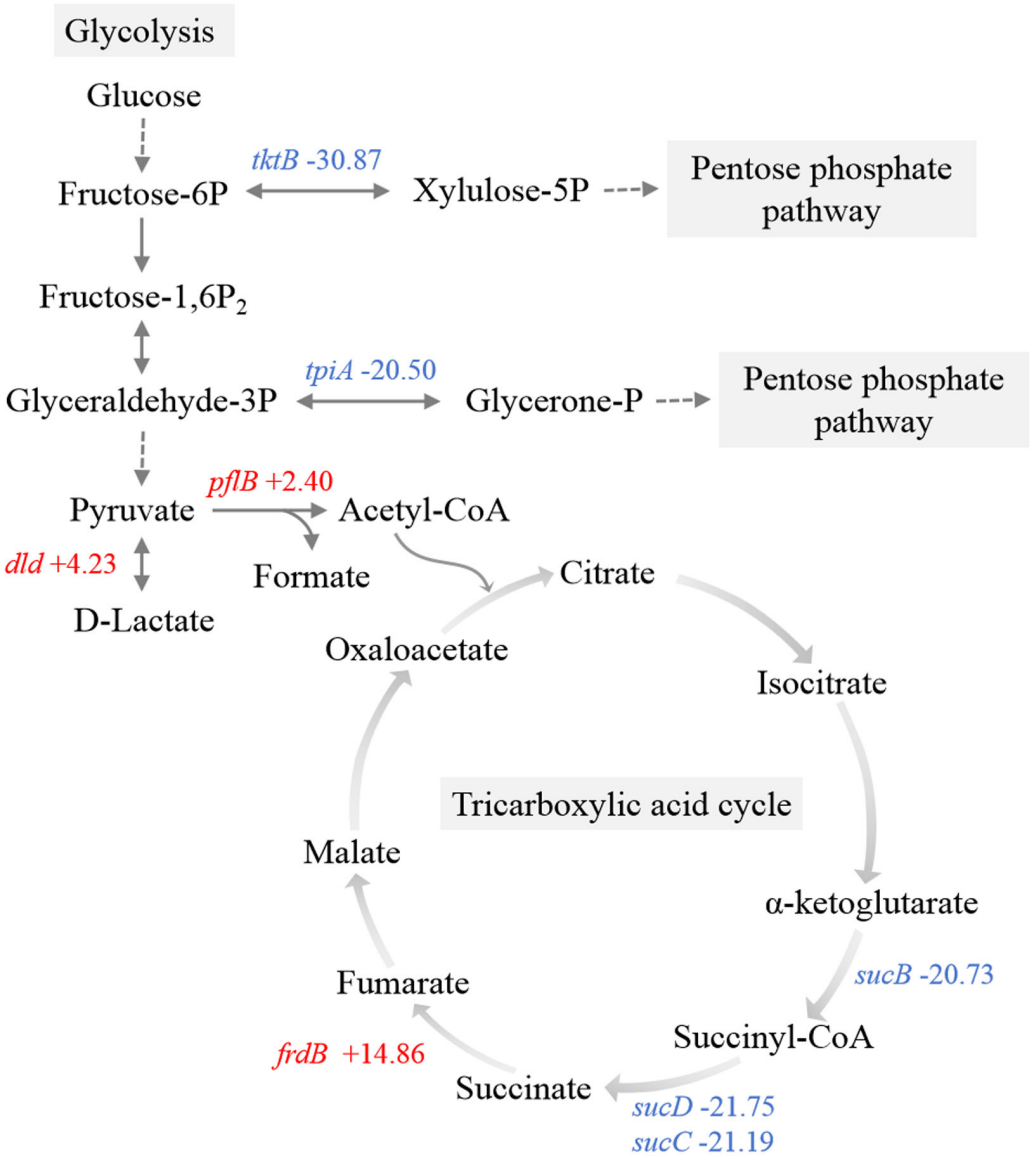
Schematic representation of the main carbon metabolism pathways. Red, upregulated genes. Blue, downregulated genes.

Transketolase and triosephosphate isomerase directly convert fructose-6-phosphate and glyceraldehyde-3-phosphate to xylulose-5-phosphate and glycerone-1-phosphate, respectively, serving as pivotal enzymes associated with glycolysis *via* the PPP (Ayoola et al., 2019). The genes encoding transketolase (*tktB*) and triosephosphate isomerase (*tpiA*) were significantly downregulated, which indicated the inhibition of the metabolic shift from glycolysis towards the PPP (**Figure 6**). Within the pyruvate metabolic network, there was an upregulation of genes encoding pyruvate formate-lyase (*pflB*) and D-lactate dehydrogenase (*dld*).

Observations included the downregulation of a component (*sucB*) associated with the 2-oxoglutarate dehydrogenase complex (**Figure 6**). Furthermore, there was significant downregulation of succinyl-CoA synthetase (*sucC* and *sucD*), which is responsible for the sole substrate-level phosphorylation step in the TCA cycle, indicating a restricted rate of the entire TCA cycle and ATP production. Additionally, transcriptome data revealed the upregulation of *frdB*, a gene encoding the iron-sulfur subunit of fumarate reductase, which catalyzes the conversion of fumaric acid to succinic acid in the TCA cycle.

### DEGs of functional proteins

In addition to the DEGs implicated in central metabolic pathways, other identified DEGs play crucial roles in the physiological metabolic processes of bacteria. These include four ribosomal subunit proteins (*rplD*, *rplV*, *rplP*, and *rpsE*), translation elongation factor EF-Tu (*tuf1*), and translation initiation factor IF-3 (*infC*) (**Supplementary Table S5**).

The ten DEGs involved in the biosynthesis of secondary metabolites are summarized in **Table 3**. The upregulated genes included glycerol-3-phosphate dehydrogenase (*glpA*), acetylglutamate kinase (*argB*), N-acetylglutamylphosphate reductase (*argC*), tryptophanase (*tnaA*), and L-asparaginase (*ansB*). The downregulated genes included catalase (*katE*), acyl carrier protein (*acpP*), proline dehydrogenase (*putA*), heme o synthase (*cyoE*), and ketol-acid reductoisomerase (*ilvC*). Additionally, fourteen DEGs previously implicated in host adaptation are summarized and listed in **Table 4**. These genes include stress response protein (*hdeA*), virulence factors (*ompF*, *cvaC*, and *hlyF*), and essential genes required for mouse and duck models of *E. coli*-related septicemia (*wecB*, *cyoB*, *frdB*, *slyD*, *dcuA*, and *hupA*) (Zhang et al., 2019).

**Table 3:** Differentially expressed genes involved in the biosynthesis of secondary metabolites.

Gene locus tag	Gene	KEGG ID	Description	Fold change	FDR	Metabolite
OI124_01840	*glpA*	K00111	Glycerol-3-phosphate dehydrogenase	5.73	0.0022	Glycerone-P, sn-Glycerol-3P
OI124_04745	*katE*	K03781	Catalase	-19.32	0.0249	O_2_
OI124_07875	*acpP*	K02078	Acyl carrier protein	-22.24	0.0444	2-Methylquinolin-4-ol
OI124_08270	*putA*	K13821	Proline dehydrogenase	-18.45	0.0276	L-Glutamate
OI124_11515	*cyoE*	K02257	Heme o synthase	-20.82	0.0198	Heme O
OI124_15960	*argB*	K00930	Acetylglutamate kinase	6.15	0.0316	N-Acetyl-glutamyl-P
OI124_15965	*argC*	K00145	N-acetylglutamylphosphate reductase	9.73	9.74E-07	N-Acetylglutamate semialdehyde
OI124_17115	*ilvC*	K00053	Ketol-acid reductoisomerase	-20.03	0.0210	(S)-2-Acetolactate, et al.
OI124_17460	*tnaA*	K01667	Tryptophanase	5.37	0.0048	Indole
OI124_21890	*ansB*	K01424	L-asparaginase	5.45	3.48E-06	L-Aspartate

**Table 4:** Differentially expressed genes involved in host adaptation.

Gene locus tag	Gene	Description	Fold change	FDR	Functions in host adaptation	Reference
OI124_18540	*hdeA*	Stress response protein	-5.13	1.56E-06	Acid-resistance; Temperature response	(Bauchart et al., 2010)
OI124_18560	*slp*	Outer membrane lipoprotein	-22.19	0.0030	Starvation protein; Temperature response	
OI124_04745	*katE*	Catalase	-19.32	0.0249	Temperature response	
OI124_08705	*ompF*	Outer membrane porin	4.52	0.0272	Virulence factor; Temperature response	
OI124_18285	*cspA*	RNA chaperone and antiterminator	-23.04	0.0146	Cold shock regulator	
OI124_17115	*ilvC*	Ketol-acid reductoisomerase	-20.03	0.0210	Temperature response	
OI124_25160	*cvaC*	Colicin V	-10.92	0.0393	Virulence factor	(Kathayat et al., 2021)
OI124_25335	*hlyF*	Hemolysin F	-19.99	0.0416	Virulence factor	
OI124_03035	*wecB*	UDP-N-acetylglucosamine 2-epimerase	-22.49	0.0185	Essential gene during infection in duck blood	(Zhang et al., 2019)
OI124_11500	*cyoB*	Cytochrome o ubiquinol oxidase subunit I	-20.29	0.0043	Essential gene during infection in duck blood	
OI124_14895	*frdB*	Fumarate reductase iron-sulfur subunit	14.86	0.0207	Essential gene during infection in duck blood	
OI124_19350	*slyD*	Peptidylprolyl isomerase	-15.23	0.0256	Essential gene during infection in duck blood	
OI124_14980	*dcuA*	Anaerobic C4-dicarboxylate transporter	3.77	0.0181	Essential gene during infection in both mouse and duck blood	
OI124_15745	*hupA*	Transcriptional regulator	-22.41	0.0248	Essential gene during infection in both mouse and duck blood	

### Differentially expressed sRNAs

sRNAs play a vital role in pathogen−host interactions among pathogenic Enterobacteriaceae (Sauder and Kendall, 2021). To identify sRNAs responsive to APEC colonizing host tissues, we analyzed significantly differentially expressed sRNAs in the transcriptome data. A total of 120 sRNAs were detected and are listed in **Supplementary Table S7**. Among these sRNAs, 7 were upregulated, and the remaining 113 were downregulated. However, only 14 of the identified sRNAs were annotated from the sRNAMap database. Known sRNAs, including SroC, naRNA4, CsrC, GlmY_tke1, GadY, and STAXI, were downregulated.

## Discussion

In this study, the virulent APEC O145 strain NC22 was selected for whole-genome sequencing, pathogenicity testing, and transcriptome analysis *in vivo*. We have investigated the prevalence and antimicrobial resistance profile of bacterial pathogens isolated from poultry in Jiangxi Province, China. The results showed that APEC was the most prevalent pathogenic bacterial species with high levels of multidrug-resistance, especially in duck flocks (Tan et al., 2023). Subsequent genomic analysis proved that O145 is the most dominant serotype (unpublished data), which is consistent with the result of the recent epidemiologic investigation (Wang et al., 2022). Moreover, understanding the genetic and molecular basis of pathogen−host interactions is crucial for elucidating its pathogenic mechanisms and designing new strategies and therapeutics for controlling APEC infections (Zhang et al., 2019). Therefore, our results can provide novel insights into processes that are important for the pathogenesis and host adaptation mechanism of APEC O145.

Avian colibacillosis is characterized by multisystem organ lesions, notably pericarditis, perihepatitis, and peritonitis (Joseph et al., 2023). The study confirmed the presence of these three typical symptoms through necropsy examination. Besides, we investigated the colonization patterns of NC22 in both blood and tissues and observed extensive tissue damage resulting from rapid bacterial replication. Pathohistological observation revealed that bacterial colonization caused severe damage to the heart, liver, spleen, lung, and kidney of infected ducks. Bacterial attacks were observed in the duodenum, jejunum, and cecum, while the ileum and rectum did not appear to be target tissues for NC22. Previous research has demonstrated the ability of APEC to invade duck brain microvascular endothelial cells and breach the blood−brain barrier (Li et al., 2016). In this study, bacterial colonization was detected in brain samples, indicating that NC22 can invade the brains of infected ducks. However, no lesions were observed in the brain parenchyma, suggesting that the bacteria may only colonize the meninges.

Central metabolic pathways of pathogenic bacteria are required for host fitness. Several genes involved in central metabolism were significantly upregulated after APEC O1 was transferred from LB to chicken serum, including *tktB* (Li et al., 2011). By contrast, metabolic pathway analysis revealed a limited flow of carbohydrates through the main glycolytic pathway to the PPP in this study. The PPP generates NADH and NADPH to fulfill the bacterial demand for reducing power (Nakamya et al., 2021). The significant downregulation of *sucB*, sucC, and *sucD* not only directly limits the rate of the TCA cycle but also hampers the production of NADH and NADPH. A higher NAD^+^/NADH ratio can protect cellular macromolecules, such as nucleic acids and proteins, from stresses (Tan et al., 2022). Moreover, previous studies have shown that compared with wild-type strains, *E. coli* strains lacking TCA cycle-related genes exhibit longer stationary-phase survival and increased resistance to heat and superoxide stresses (Gonidakis et al., 2010). Accordingly, it is hypothesized that inhibiting the PPP and TCA cycle is necessary for the maximum lifespan extension of *E. coli* colonizing host tissue. Bacteria likely have a greater demand for the NAD^+^/NADH and NADP^+^/NADPH ratios than bacteria cultured *in vitro* due to environmental stresses, such as high temperature and superoxide released by host immune cells. The findings also suggest that pathogenic bacteria employ different survival strategies in response to different host ecological niches. Further metabolomics analysis targeting the bacteria in the host will help validate our hypothesis and elucidate the underlying molecular mechanisms that drive changes in the central metabolic pathways.

DEGs involved in the biosynthesis of secondary metabolites were identified (**Table 3**). These metabolites play important physiological roles. For instance, the conversion of tryptophan to indole by the enzyme tryptophanase (*tnaA*) is a signaling event that triggers various physiological changes in *E. coli*. Indole also regulates interactions between bacteria and their hosts, including virulence, biofilm formation, motility, plasmid stability, and acid resistance (Li and Young, 2015). Furthermore, indole functions as a logarithmic-phase signal molecule (Kobayashi et al., 2006). The upregulation of *tnaA* suggested that the bacteria in the liver at 2 dpi still reproduced rapidly, consistent with the bacterial colonization pattern observed in tissues. Another significant enzyme, catalase (*katE*), is responsible for combating oxidative stress by removing reactive oxygen species in *E. coli* (Wan et al., 2017). Surprisingly, *katE* was found to be significantly downregulated in this study, but the specific mechanism underlying this downregulation remains unclear.

This study revealed 14 DEGs that have been reported in previous *in vitro* stress assays and poultry infection models (**Table 4**). Among the 14 DEGs, *hdeA*, *slp*, *katE*, *ompF*, *cspA*, and *ilvC* are regulated genes in an APEC O18 strain in response to 41°C versus 37°C (Bauchart et al., 2010). Moreover, *wecB*, *cyoB*, *frdB*, and *slyD* are virulence-essential genes identified in the avian model of *E. coli*-related septicemia, while *dcuA* and *hupA* are shared virulence-essential genes associated with systemic infection in both mammalian and avian models of *E. coli*-related septicemia (Kathayat et al., 2021). These stress response factors and essential virulence genes with significantly changed transcriptional levels were observed in NC22 colonizing *in vivo*, which suggesting a similar behavior of these APEC strains against challenging survival environments.

In recent years, much attention has been given to the roles of sRNAs in bacterial species, particularly *E. coli* and *Salmonella* spp., as part of regulatory responses to environmental stress (Hor et al., 2020). At 2 dpi, NC22 colonization in the duck liver led to the differential expression of numerous sRNAs, including SroC, CsrC, and GadY. SroC, which originates from the *gltI*-*gltJ* intergenic region, controls the levels of GcvB, a base-pairing RNA. The interaction between GcvB and SroC directs the cleavage of GcvB by RNase E (Miyakoshi et al., 2015). CsrC sequesters and reduces the activity of CsrA, which plays a key role in modulating the carbon storage regulatory system and pathogenesis (Pourciau et al., 2019). Therefore, the downregulation of CsrC would enhance CsrA activity. GadY, an Hfq-binding sRNA, has been observed to downregulate the antisense-encoded *gadXW* mRNA by directing RNase III-mediated cleavage (Opdyke et al., 2011). However, given the relatively young state of the field of bacterial sRNAs, the specific mechanisms and physiological processes regulated by multiple novel sRNAs remain unknown. The differentially expressed sRNAs identified in this study can provide a foundation for further studies in the coming years, which are expected to uncover new mechanisms and answers related to sRNAs.

In conclusion, this study aimed to elucidate the mechanisms associated with APEC O145-related pathogenicity and host adaptation. This study provides experimental evidence for understanding the patterns of damage and colonization caused by the APEC O145 strain in duck blood and tissues. Transcriptome analysis of APEC O145 revealed DEGs and differentially expressed sRNAs that play crucial roles in bacterial infection in the host liver. However, the functions of most identified DEGs and sRNAs have been unclear. The underlying mechanisms of the downregulation of most DEGs, sRNAs, and virulence genes remain unknown. Moreover, it is unclear whether the findings in this study can be generalized to other APEC O145 strains. Consequently, further investigations are urgently warranted to enhance our comprehensive understanding of the mechanisms governing APEC-specific host-pathogen interactions.

## Data Availability

The datasets presented in this study can be found in online repositories. The names of the repository/repositories and accession number(s) can be found in the article/**Supplementary Material**.

## Supplementary material

Supplementary Figure 1 Clinical dissection and observation of NC22-challenged ducks. The yellow arrow s represent the fibrous exudate on the heart, liver, and periphery.

Supplementary Figure 2 Correlation analysis between RNA sequencing data and qRT−PCR assay results.
