# Supplementary figures and images for: Pathogenicity and identification of host adaptation genes of the avian pathogenic Escherichia coli O145 in duck
Source: Front Cell Infect Microbiol. 2024 Nov 13;14:1453907. doi: 10.3389/fcimb.2024.1453907 (PMC11599210; doi:10.3389/fcimb.2024.1453907)

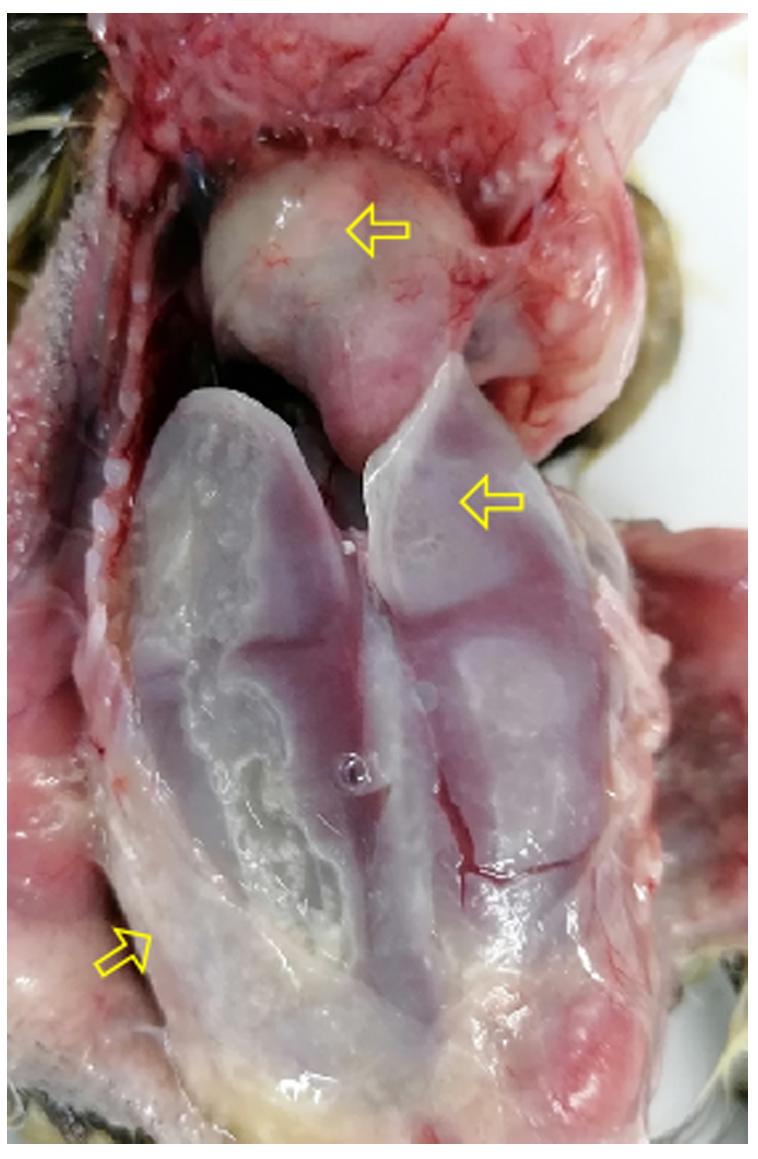

Supplement: Supplementary Figure 1 — Clinical dissection and observation of NC22-challenged ducks. The yellow arrow s represent the fibrous exudate on the heart, liver, and periphery. [file Image1.tif]

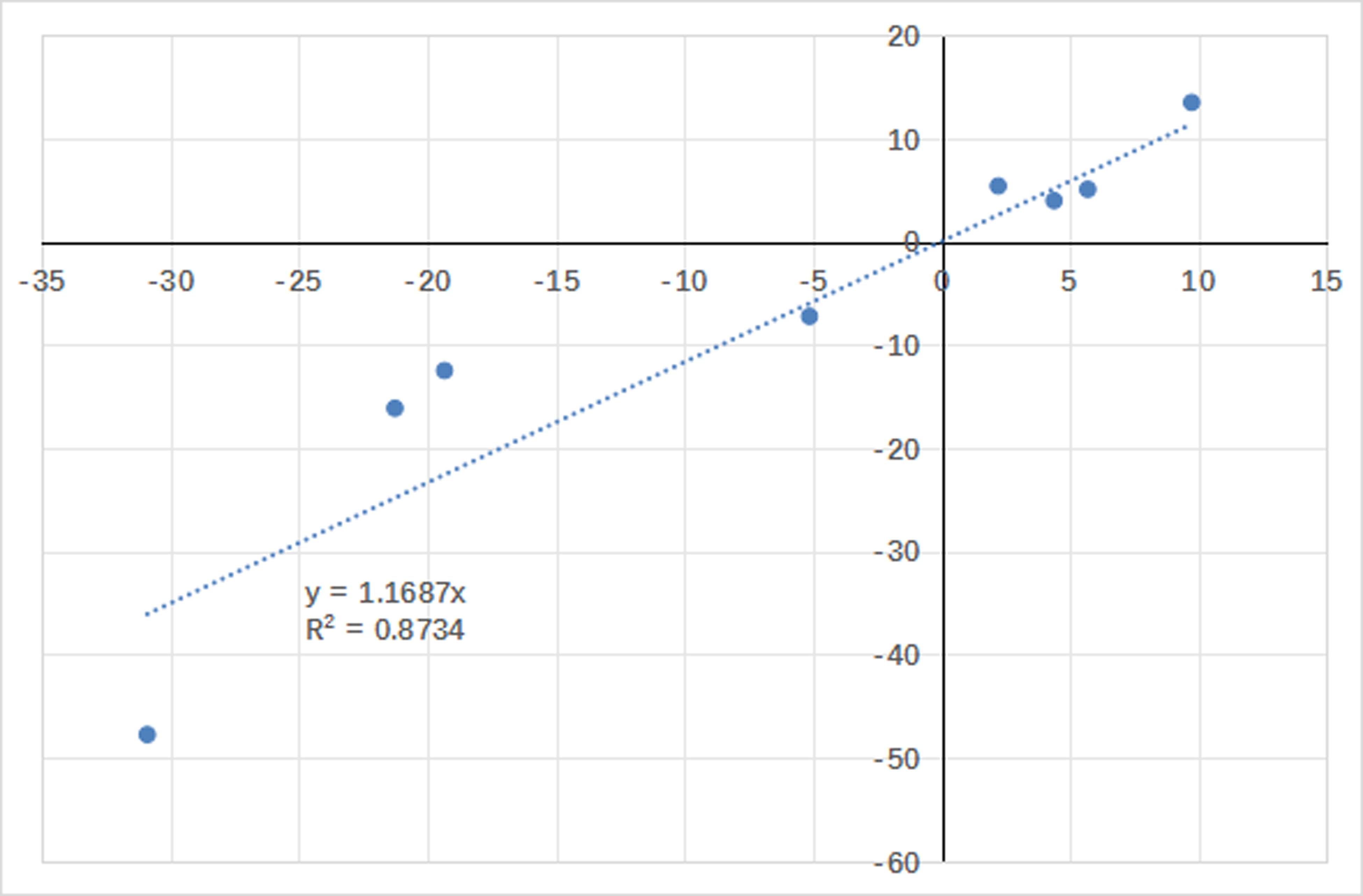

Supplement: Supplementary Figure 2 — Correlation analysis between RNA sequencing data and qRT−PCR assay results. [file Image2.tif]
